# One-step facile synthesis of nickel–chromium layered double hydroxide nanoflakes for high-performance supercapacitors†

**DOI:** 10.1039/d0na00178c

**Published:** 2020-04-24

**Authors:** Zuo Chen, Hao Deng, Man Zhang, Zhiyu Yang, Di Hu, Yuchen Wang, Kai Yan

**Affiliations:** School of Environmental Science and Engineering, Sun Yat-sen University 135 Xingang Xi Road Guangzhou 510275 P. R. China yank9@mail.sysu.edu.cn; Guangdong Provincial Key Laboratory of Environmental Pollution Control and Remediation Technology Guangzhou 510275 P. R. China

## Abstract

Rational design and synthesis of efficient electrodes with pronounced energy storage properties are crucial for supercapacitors. Herein, we report thin NiCr-layered double hydroxide nanoflakes (NiCr-LDNs) for a high-performance supercapacitor. These fabricated NiCr-LDNs, with various Ni^2+^/Cr^3+^ ratios, are one-step controllably synthesized through ultrasonication coupled with mechanical agitation, without hydrothermal treatment or extra exfoliation using organic solvents. Through comparison of different Ni^2+^/Cr^3+^ ratios, the Ni_2_Cr_1_-LDNs with a 4.7 nm thickness exhibited a superb capacitance performance of 1525 F g^−1^ at 2 A g^−1^, which is competitive with most previously reported layered double hydroxide (LDH)-based electrodes. These thin nanoflake structures have the potential to reduce the energy barrier and enhance the capture ability of electrolyte ions. Besides, an asymmetric supercapacitor (ASC) assembled using Ni_2_Cr_1_-LDNs achieved a remarkable energy density of 55.22 W h kg^−1^ at a power density of 400 W kg^−1^ and maintained high specific capacitance (over 81%), even after 5000 cycles. This work offers an efficient and facile route to fabricating LDH nanoflakes for boosting energy storage capabilities.

## Abstract 2

Rational design and synthesis of efficient electrodes with pronounced energy storage properties are crucial for supercapacitors.

## Graphical abstract

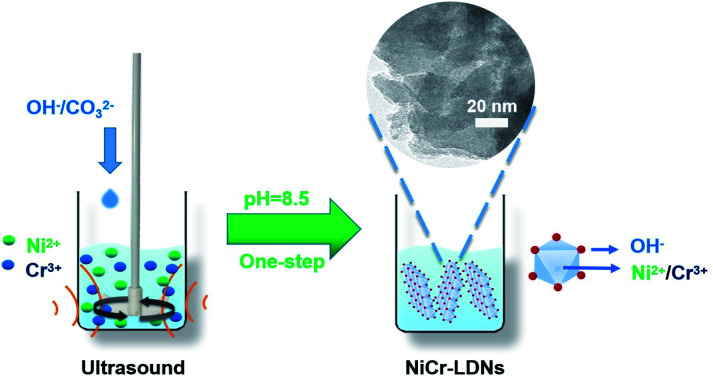

## Introduction

Layered double hydroxides (LDHs), also called hydrotalcite-like materials, are a family of two-dimensional anionic compounds formed by the orderly arrangement of anions between layers and positively charged laminates.^1,2^ The formula of LDHs is expressed as follows: M_1−*x*_^2+^M_*x*_^3+^(OH)_2_(A^*n*−^)_*x*/*n*_·*m*H_2_O, where M^2+^, M^3+^ and A^*n*−^ represent divalent metal ions, trivalent metal ions and intercalated anions, respectively.^1–6^ LDHs are alkaline and thermally stable, can exchange anions, and have a controllable composition and structure.^7–9^ On the one hand, the unique spatial structure of LDHs provides a large surface area to transfer charge and capture electrons.^8,10^ On the other hand, the high dispersion of variable valence metal ions on the lamella can provide abundant electrochemically active sites, resulting in high pseudocapacitance.^11–13^ Over the past few decades, LDHs have attracted considerable attention for use as electrochemical supercapacitors. For example, Liu *et al.* fabricated NiO/NiMn-LDHs by a facile two-step approach and reported a specific capacitance of 937 F g^−1^ at a current density of 0.5 A g^−1^.^14^ Li *et al.* fabricated NiFe-LDHs/graphene for use as a supercapacitor with a good specific capacitance of 1462.5 F g^−1^ at 5 A g^−1^.^15^ Zhang *et al.* synthesized a NiAl-LDH pseudo-capacitor electrode, which exhibited a specific capacitance of 1.040 C cm^−2^ at 1.68 mA cm^−2^.^16^

However, LDHs still have their own intrinsic shortcomings, for example, the accumulation of layers and the blocked interlayer space limit electrolyte ion access to internal active sites.^17,18^ In order to facilitate the diffusion of ions and enhance electronic conductivity, researchers have developed several fabrication strategies to reduce the thickness of layers and increase the distance between layers. One of the most common methods is chemical stripping. In a pioneering study, Hu *et al.* investigated the synthesis of CoCo-, NiFe- and NiCo-LDH nanosheets by dispersing LDH powder into 100 mL of formamide and stirring for 24 h.^19^ Zhong *et al.* studied the exfoliation of CoAl-LDHs by stirring for 2 days in formamide.^20^ Du *et al.* synthesized NiAl-LDHs in a formamide solution by stirring for 3 days under nitrogen protection.^20^ In addition, some new exfoliation methods (*e.g.*, Ar-plasma, nitrogen-plasma, and solid-phase exfoliation techniques) were used to synthesize thin LDHs.^17,22,23^ Our previous studies have also shown that ultrasonication-assisted hydrothermal synthesis is a good approach to fabricate thin CoMn-LDHs and NiCo-LDHs in several steps, enhancing the catalytic performance of oxygen evolution in comparison with bulk LDH precursors.^17,24^ However, these exfoliation methods still have several steps and, therefore, suffer from low efficiency and complexity, are time-consuming and require a large amount of chemical reagents. Besides, the energy storage performance and durability of the previously reported LDHs still need to be improved.^17,24^ Developing a one-step, facile method to fabricate thin LDHs with a controllable morphology for boosting energy storage capacity is still a challenge.

Inspired by the advanced studies, we endeavored to control the synthesis of thin NiCr-layered double hydroxide nanoflakes (NiCr-LDNs) with different Ni^2+^/Cr^3+^ ratios in one step, and these fabricated LDNs served as a superior pseudocapacitive electrode for energy storage. This alternative construction strategy shows great potential for the large-scale production of nanoflakes. Several types of NiCr-LDNs with different Ni^2+^/Cr^3+^ ratios were successfully designed and evaluated with electrochemical tests. The Ni_2_Cr_1_-LDN electrode achieved a remarkable specific capacitance of 1525 F g^−1^ at a current density of 2 A g^−1^ in a 6 M KOH electrolyte using the three-electrode system. Besides, the Ni_2_Cr_1_-LDNs were used as a positive electrode to assemble an asymmetric supercapacitor, which provided a relatively outstanding energy density of 55.22 W h kg^−1^ at a power density of 400 W kg^−1^, retaining 81.1% of the original specific capacitance even after 5000 cycles. Furthermore, Ni_2_Cr_1_-LDNs exhibited low resistance, fast kinetics and long durability. This work provides a facile and efficient way to fabricate LDH nanoflakes to largely enhance capacitance.

## Experimental

### Synthesis of NiCr-LDNs

**Fig. 1:**
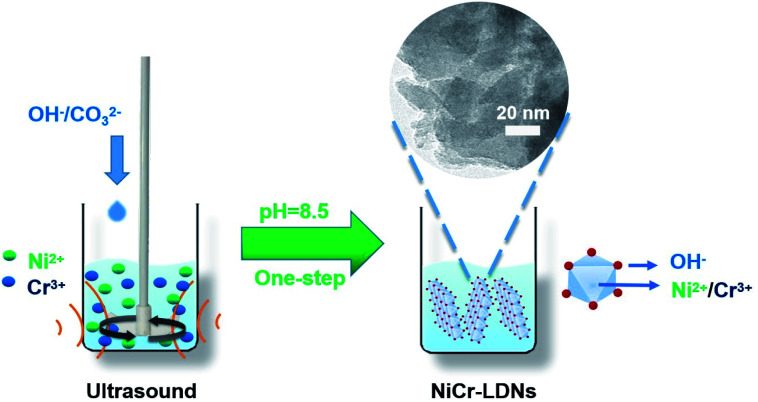
Schematic illustration of the facile synthesis of NiCr-LDNs in one-step.

All of the chemicals were used without any purification. Nickel chloride hexahydrate (NiCl_2_·6H_2_O), chromium chloride hexahydrate (CrCl_3_·6H_2_O), sodium hydroxide (NaOH), and sodium carbonate (Na_2_CO_3_) were bought from Macklin Chemical Reagent Co. Ltd. The general synthesis procedure is depicted in Fig. 1. NiCl_2_·6H_2_O and CrCl_3_·6H_2_O were dissolved in distilled water to prepare 1 M aqueous solutions. Then, NiCl_2_ (1 M) and CrCl_3_ (1 M) were added to a beaker to achieve mixed solution A (10 mL, the molar ratios of Ni^2+^ and Cr^3+^ in solution A ranged from 3 : 1 to 1 : 2). Solution A was mechanically stirred (200 rpm) and ultrasonicated (40 kHz) simultaneously. About 0.2 mol of Na_2_CO_3_ and 0.8 mol of NaOH were dissolved in distilled water (1 L), and then the mixture (solution B) was slowly titrated into solution A to adjust the pH to 8.5 and held for 1 h. After centrifugation and vacuum drying at 50 °C for 14 h, the powder products were collected and denoted as Ni_3_Cr_1_-LDNs, Ni_2_Cr_1_-LDNs, Ni_1_Cr_1_-LDNs, and Ni_1_Cr_2_-LDNs corresponding to a Ni^2+^/Cr^3+^ ratio of 3 : 1, 2 : 1, 1 : 1, and 1 : 2, respectively. The reference Ni_2_Cr_1_-LDHs were synthesized in the same way without ultrasonic treatment.

### Electrode characterization

X-ray diffraction (XRD) characterization was conducted on a D/max-2200vpc (Rigaku, Japan) at 40 kV and 26 mA with Cu Kα_1_ radiation of 1.54060 Å. The 2-theta degree ranged from 5° to 70°, and the scan rate was 10° min^−1^. Transmission electron microscopy (TEM) was used to identify the microstructure and the morphologies of the samples (coated on Cu grids) and was performed using a JEM-2100F (JEOL, Japan) operated at 200 kV. Scanning electron microscopy (SEM, ZEISS, SIGMA 500, GER) and corresponding energy-dispersive X-ray spectrometry (EDS, Bruker, GER) were used to characterize the microstructures and element distribution of the products at 10 kV. X-ray photoelectron spectroscopy (XPS) analysis was carried out using an ESCALab 250 imaging X-ray photoelectron spectrometer system (Thermo Scientific, USA) with monochromatic Al Kα X-rays as the excitation source (*E* = 1486.6 eV) and a pass energy of 20 eV. Atomic force microscopy (AFM) was used to obtain the thickness of samples and was performed on a Dimension Edge (Bruker, USA) in tapping mode. For AFM, the samples were dispersed in an ethanol solvent and dripped onto a mica plate for testing. N_2_ adsorption–desorption isotherms of the samples were analyzed by using a Micromeritics ASAP 2020 nitrogen adsorption apparatus (USA) at 77 K, whereas the physical activation was performed at 150 °C for 10 h before measurements. The specific surface area was obtained from the adsorption isotherm curve. Pore size distributions were calculated using the Barrett–Joyner–Halenda (BJH) method, for both the adsorption and desorption curves. The total volume was estimated from the adsorbed amount at a relative pressure *P*/*P*_0_ of 0.995.

### Electrochemical characterization and tests

Galvanostatic charge–discharge (GCD), cyclic voltammetry (CV) and electrochemical impedance spectroscopy (EIS) tests of the samples were conducted using a CHI760E electrochemical workstation with an alkaline electrolyte (6 M KOH) in a three-electrode system. A platinum filament (CHI115) and saturated calomel electrode (SCE, CHI150) worked as the counter and reference electrodes, respectively. Then 70 wt% active materials, a 15 wt% polytetrafluoroethylene emulsion (PTFE, Aladdin) and 15 wt% supercarbon (Alfa Aesar) were mixed to prepare the working electrode. The mixture was then stirred and pressed onto nickel foam (1 cm^2^) under 10 MPa and dried at 70 °C for 12 h. The loaded mass of the active material was controlled at 3–4 mg cm^−2^. The specific capacitance of the electrode material was calculated using the formula:^25,26^1
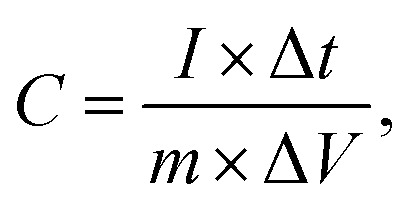
where *I*, Δ*V*, Δ*t*, and *m* represent the discharge current (A), the voltage window (V), the discharge time (s) and the quantity of the active material (g), respectively.

### Assembly and test of the asymmetric supercapacitor

The asymmetric supercapacitor was fabricated using Ni_2_Cr_1_-LDNs as the positive electrode, active carbon (AC, Kuraray) as the negative electrode, and cellulose paper as the separator in a 6 M KOH electrolyte. The preparation of the AC electrode was similar to that of the Ni_2_Cr_1_-LDN electrode. The energy density (*E*, W h kg^−1^) and power density (*P*, W kg^−1^) of Ni_2_Cr_1_-LDNs//AC were estimated according to the following equations:^15,21^2
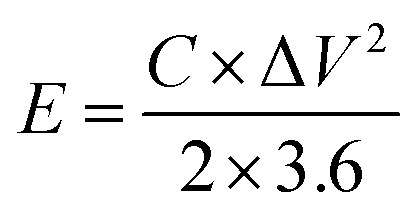
3
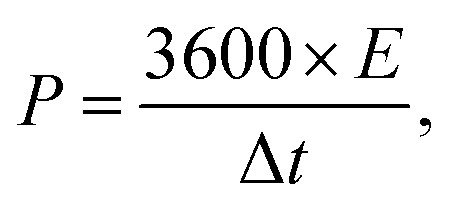
where Δ*V*, Δ*t* and *C* represent the potential window (V), the discharge time (s) and the capacitance (F g^−1^), respectively.

## Results and discussion

### Structural and morphological characterization

The crystal structures of the five materials with different Ni^2+^/Cr^3+^ ratios were identified by powder X-ray diffraction. Fig. S1† shows a series of crystal planes (003), (006), (012) and (110) of the Ni_2_Cr_1_-LDN samples at 11°, 23°, and 35° that were indexed to the characteristic peaks of LDHs synthesized without ultrasonication (PDF#89-7111). In the one-step synthesis by ultrasonication coupled with mechanical stirring, the typical layers of LDHs were exfoliated into thin nanoflakes with lower intensity and broader peaks, as indicated by the XRD patterns.^27–29^

Fig. S2† presents the morphology differences between the Ni_2_Cr_1_-LDHs and Ni_2_Cr_1_-LDNs by SEM. After the ultrasonication treatment, brucite-like layers of Ni_2_Cr_1_-LDHs were exfoliated into fragments with thin nanoflakes. The structure with thin nanoflakes could facilitate the diffusion of electrolyte ions into internal active sites to provide significant capacitance. Moreover, the corresponding EDS mapping confirmed that the Ni and Cr elements were well dispersed in Ni_2_Cr_1_-LDNs (Fig. S3†), showing the effect of ultrasonication during co-precipitation.

**Fig. 2:**
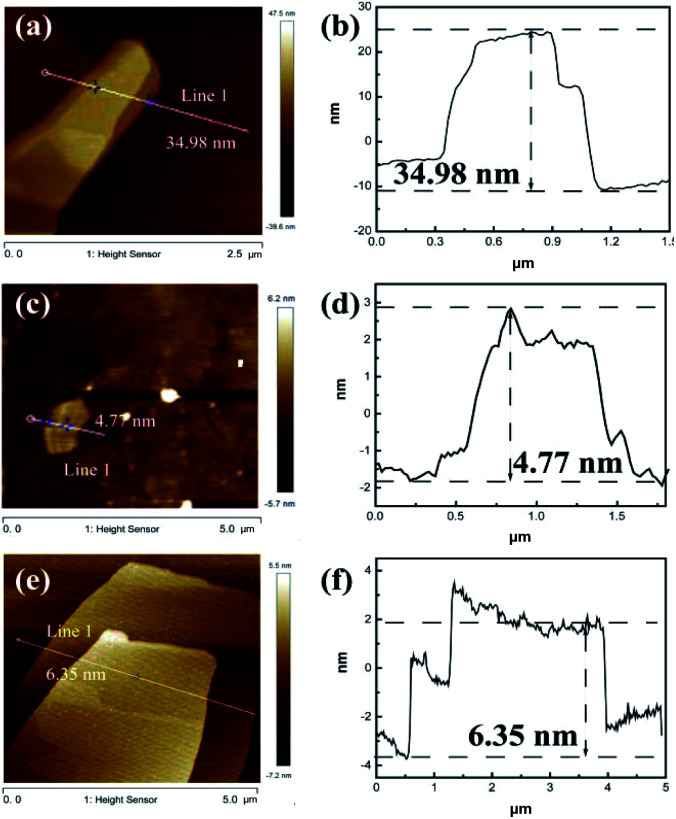
AFM images and height profile of (a and b) Ni_2_Cr_1_-LDHs, (c and d) Ni_2_Cr_1_-LDNs, and (e and f) Ni_3_Cr_1_-LDNs.

**Fig. 3:**
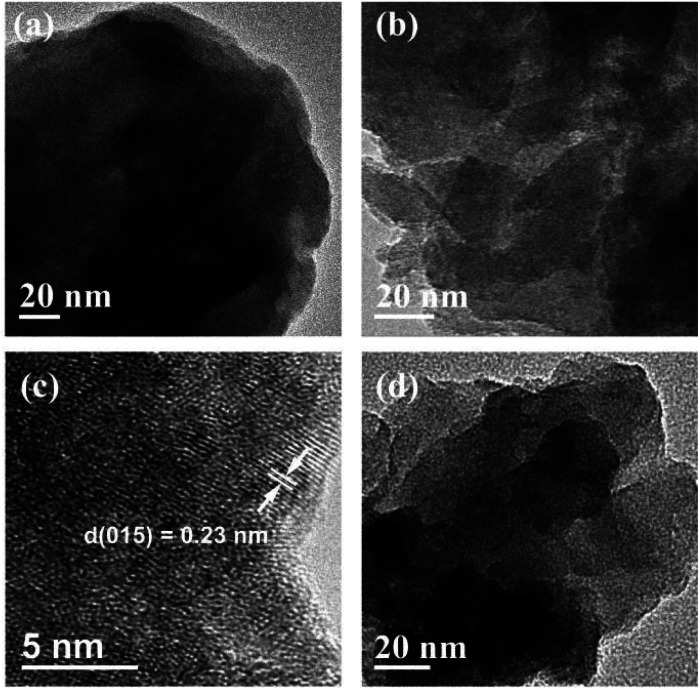
TEM images of (a) Ni_2_Cr_1_-LDHs, (b and c) Ni_2_Cr_1_-LDNs and (d) Ni_3_Cr_1_-LDNs.

AFM is a technique used to directly measure the thickness, as shown in Fig. 2, S4 and S5.† A thickness of 4.77 nm for Ni_2_Cr_1_-LDN single layer was measured, while, in comparison, the Ni_2_Cr_1_-LDHs synthesized without ultrasonication had a thickness of 34.98 nm, confirming the important role of ultrasonication in producing nanoflakes. Different NiCr-LDN nanoflakes with various Ni^2+^/Cr^3+^ ratios were also synthesized under identical conditions. Ni_1_Cr_1_-LDNs (Fig. S5†) and Ni_3_Cr_1_-LDNs (Fig. 2e and f) had 16.61 nm and 6.35 nm thicknesses, respectively. A TEM study was further conducted to analyze the microstructure of the as-prepared samples. From Fig. 3, it is clear that bulk Ni_2_Cr_1_-LDHs had several stacked layers, while the Ni_2_Cr_1_-LDN nanoflakes were successfully synthesized with thin layers (Fig. 3b). Besides, a typical lattice spacing of 0.20 nm was indexed to the (107) plane of Ni_2_Cr_1_-LDNs (PDF#89-7111).^30,31^ By comparing Ni_3_Cr_1_-LDNs (Fig. 3d) with Ni_1_Cr_1_-LDNs (Fig. S6†), it was further confirmed that ultrasonication could efficiently peel off the bulk LDH structure and form nanoflakes. According to Table S1 and Fig. S7,† the specific surface areas of Ni_1_Cr_1_-LDNs, Ni_1_Cr_1_-LDNs and Ni_1_Cr_1_-LDNs were 62.5, 73.4, and 63.3 m^2^ g^−1^, respectively. The total pore volumes of Ni_1_Cr_1_-LDNs, Ni_1_Cr_1_-LDNs and Ni_1_Cr_1_-LDNs were 0.065, 0.073, and 0.068 cm^3^ g^−1^, respectively. The nanoflake structure provides a larger surface area and allows the flow of ions between laminates, which is favorable for energy storage.^30,31^

**Fig. 4:**
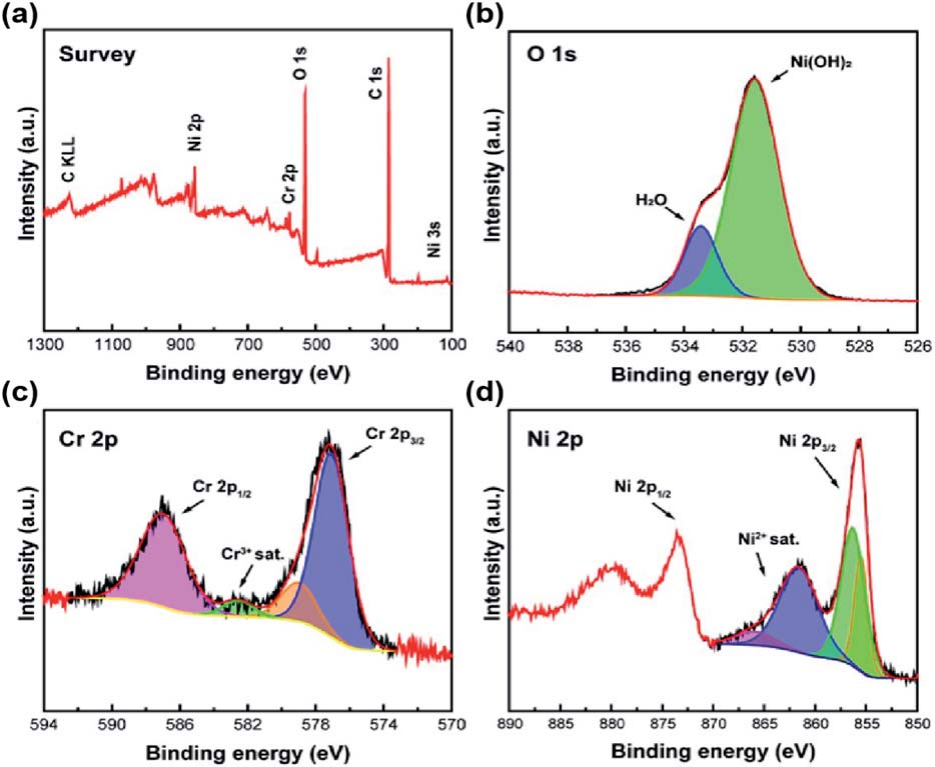
XPS spectra of Ni_2_Cr_1_-LDNs: survey (a), and fitting spectra of O 1s (b), Cr 2p (c) and Ni 2p (d).

XPS was further conducted to analyze the chemical state and composition of Ni_2_Cr_1_-LDNs. Fig. 4a shows the survey scan of the Ni_2_Cr_1_-LDNs, in which Ni, Cr, O and C elements existed. Fig. 4b illustrates that the O element is mainly in the form of Ni(OH)_2_ (531.5 eV).^32^ The O 1s lattice oxides for Cr(OH)_3_ and Cr_2_O_3_ were not distinguished due to the overlap of Ni^2+^ and Cr^3+^.^32^ From the split spin–orbit peaks of Cr 2p in Fig. 4c, it was observed that Cr(OH)_3_ can be fitted with a single 2p_3/2_ peak at 577.0 eV, and a corresponding 2p_1/2_ peak located at 586.9 eV.^33^ A lower peak at 578.9 eV was attributed to the partial oxidation of Cr^3+^. Typical Ni 2p_3/2_ peaks for Ni(OH)_2_ were fitted with the main peak (855.5 eV) and the secondary peak (856.3 eV).^32–35^ A satellite peak for Ni 2p_3/2_ was obviously displayed at 861.6 eV (Fig. 4d).^32^

### Electrochemical evaluation

The electrochemical properties of the samples were investigated through CV, GCD, and EIS. In order to study the capacitive performance of the NiCr-LDN electrodes, CV and GCD curves of the pure nickel foam electrode, Cr(OH)_3_ electrode, Ni(OH)_2_ electrode, and NiCr-LDN electrode are shown in Fig. S8a–c.† Obvious redox peaks were observed in the CV curves of the Ni(OH)_2_ electrode and the NiCr-LDN electrode. The equilibrium potential of the redox peaks was 0.3–0.4 V *vs.* SCE, which could be the faradaic redox reaction of Ni(OH)_2_ as follows:^36,37^

4 Ni(OH)_2_ + OH^−^ ↔ NiOOH + H_2_O + e^−^.

The result indicates that Ni(OH)_2_ contributed most to the capacitance of the NiCr-LDN electrode, whereas pure nickel foam and Cr(OH)_3_ contributed very little. Furthermore, compared with the Ni(OH)_2_ electrode, the higher current density of the redox peaks of the NiCr-LDN electrode clearly demonstrates that interactions between Ni and Cr could improve the electrochemical performance. According to Fig. S8c,† the specific capacitance of the Ni_2_Cr_1_-LDN electrode was 1525 F g^−1^, which was higher than that of the Ni(OH)_2_ electrode (812 F g^−1^).

**Fig. 5:**
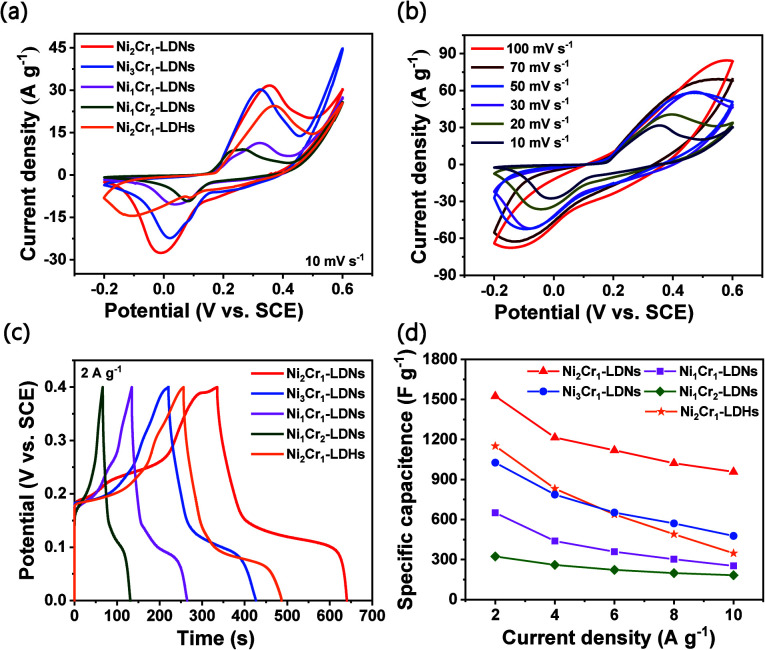
(a) CV curves of LDN and LDH electrodes at a scan rate of 10 mV s^−1^, (b) CV curves of Ni_2_Cr_1_-LDNs at various scan rates from 5 to 100 mV s^−1^, (c) GCD curves of LDNs and LDHs at a current density of 2 A g^−1^, and (d) specific capacitances of LDN and LDH electrodes at different current densities.

The CV curves of the Ni_2_Cr_1_-LDN, Ni_3_Cr_1_-LDN, Ni_1_Cr_1_-LDN, Ni_1_Cr_2_-LDN, and Ni_2_Cr_1_-LDH electrodes at 10 mV s^−1^ in the potential range of −0.2 to 0.6 V are compared in Fig. 5a. Notably, Ni_2_Cr_1_-LDNs exhibited much higher redox peaks than the other four samples, illustrating that Ni_2_Cr_1_-LDNs possess the highest specific capacitance. In addition, the reduction peak of Ni_2_Cr_1_-LDHs shifted to a lower potential due to the stacking of layers, which affects the transfer of ions and charge.^38^Fig. 5b displays the CV curves of Ni_2_Cr_1_-LDNs at various scan rates. The anodic peak shifted to a positive potential and the cathodic peak shifted to a negative potential as the scan rate increased because the Ni_2_Cr_1_-LDN electrode is quasi-reversible and polarizable. According to the CV curves, the apparent heterogeneous electron transfer rate constant (*k*_s_) was estimated using the formula:^39,40^5
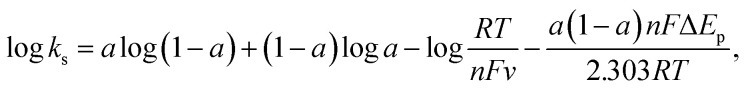
where Δ*E*_p_ is the peak potential separation, *n* represents the number of electrons transferred in the faradaic reaction, *v* represents the scan rate, and *T*, *R*, *F*, and *a* represent constants. The average *k*_s_ value of the Ni_2_Cr_1_-LDN electrode was 0.595 cm s^−1^, which is higher than that of the Ni_2_Cr_1_-LDH electrode (0.307 cm s^−1^). These results indicated that exfoliation of the layered structure contributed to fast ion transfer.

Fig. 5c gives the GCD curves of the Ni_2_Cr_1_-LDN, Ni_3_Cr_1_-LDN, Ni_1_Cr_1_-LDN, Ni_1_Cr_2_-LDN, and Ni_2_Cr_1_-LDH electrodes at 2 A g^−1^. The LDNs had the highest discharge time when the ratio of Ni^2+^ to Cr^3+^ was 2 : 1. Fig. S8d† shows the GCD curves of the Ni_2_Cr_1_-LDN electrode at various current densities. The GCD curve had flat discharge and charge platforms, and the charge time was basically the same as the discharge time, which suggests that the electrode had good coulombic efficiency and reaction reversibility.^40^Fig. 5d presents the calculated specific capacitances of the five electrodes at various discharge current densities. When the current density was set at 2 A g^−1^, the specific capacitances of the Ni_2_Cr_1_-LDN, Ni_3_Cr_1_-LDN, Ni_1_Cr_1_-LDN, and Ni_1_Cr_2_-LDN electrodes were 1525, 1027, 651, and 324 F g^−1^. When the current density was gradually increased to 10 A g^−1^, their specific capacitances diminished correspondingly to 957, 477, 253, and 183 F g^−1^, and the capacitance retentions reached up to 62.8%, 46.4%, 41.1%, and 56.5%, respectively. The specific capacitance of Ni_2_Cr_1_-LDHs only retained 29.0% (347.5 F g^−1^) at 10 A g^−1^*versus* 1198 F g^−1^ at 2 A g^−1^, showing the minimum performance rate among the five samples. This is because the accumulation of layers blocked the flow of ions, while the partial peeling of the laminates after ultrasonication facilitated the rapid flow of ions. When the current density increased and the transfer of ions and charge sped up, the influence of the stacked laminate structure of LDHs became more obvious.

**Fig. 6:**
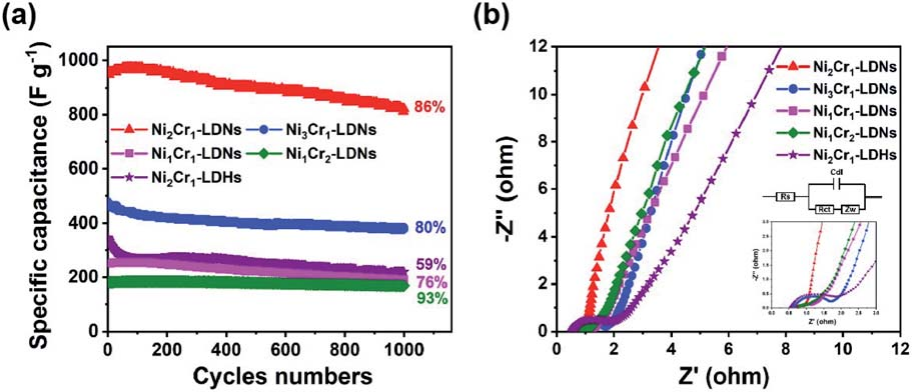
(a) Cycle stability of LDN and LDH electrodes at 10 A g^−1^, and (b) Nyquist plots of LDN and LDH electrodes.

The cyclability of the five electrodes is displayed in Fig. 6a. The capacitance retentions of the Ni_2_Cr_1_-LDN, Ni_3_Cr_1_-LDN, Ni_1_Cr_1_-LDN, Ni_1_Cr_2_-LDN, and Ni_2_Cr_1_-LDH electrodes were 86%, 80%, 76%, 93%, and 59% after 1000 cycles, suggesting that the LDNs exfoliated by ultrasound had a higher cycling stability. EIS was introduced to study the resistance of the NiCr-LDN electrodes (Fig. 6b). The diameter of the semicircle in the high-frequency range reflects the charge transfer resistance (*R*_ct_). The *R*_ct_ values of Ni_2_Cr_1_-LDNs, Ni_1_Cr_2_-LDNs, Ni_1_Cr_1_-LDNs, Ni_3_Cr_1_-LDNs and Ni_2_Cr_1_-LDHs were 0.26, 0.50, 0.64, 1.32, and 1.57 Ω, respectively, and the charge transfer resistance of Ni_2_Cr_1_-LDNs was the lowest. A low *R*_ct_ was important for enhancing the electrical conductivity and excellent rate capability. This result was associated with the reduced thickness of the layers and the increased specific surface area, which could facilitate the diffusion of electrolyte ions and fast kinetics. As listed in Table 1, the electrochemical performance of Ni_2_Cr_1_-LDNs was comparable with or superior to that of LDH-based materials in the literature. Therefore, this material is a promising electrode material for high-performance supercapacitors.

**Table 1:** Comparison of the electrochemical performance of LDH-based materials

Materials	Specific capacitance	Current density	Electrolyte	Ref.
GO/CoAl-LDHs	825 F g^−1^	1 A g^−1^	6 M NaOH	20
CoAl-LDHs	838 F g^−1^	1 A g^−1^	6 M KOH	41
NiMn-LDHs/C	1634 F g^−1^	1 A g^−1^	6 M KOH	42
NiMn-LDHs	1202 F g^−1^	5 A g^−1^	1 M KOH	12
NiCo-LDHs/GO	1489 F g^−1^	1 A g^−1^	6 M KOH	43
NiCo_2_O_4_@NiFe-LDHs	1160 F g^−1^	1 A g^−1^	2 M KOH	44
NiO/NiMn-LDHs	937 F g^−1^	0.5 A g^−1^	3 M KOH	14
NiFe-LDHs	1462.5 F g^−1^	5 A g^−1^	2 M KOH	15
CoAl-LDHs/rGO	1492 F g^−1^	1 A g^−1^	6 M KOH	22
NiMn-LDHs	733.8 F g^−1^	1 A g^−1^	1 M LiOH	45
NiMn-LDHs/Ni foam	1511 F g^−1^	2.5 A g^−1^	1 M KOH	46
NiCoAl-LDHs	1137 F g^−1^	0.5 A g^−1^	6 M KOH	7
NiCo-LDHs	1410 F g^−1^	2 A g^−1^	6 M KOH	47
CoS_*x*_/Ni–Co LDHs	1562 F g^−1^	1 A g^−1^	2 M KOH	10
Ni_2_Cr_1_-LDNs	1525 F g^−1^	2 A g^−1^	6 M KOH	This work

In general, the main reasons for the remarkable performance of the Ni_2_Cr_1_-LDNs could be attributed to the following features: first, the process of ultrasonication during co-precipitation resulted in the exfoliation of LDHs and the formation of nanoflakes.^5^ LDH nanoflakes had more active sites than the stacked LDHs, which facilitated rapid ion transport, leading to more efficient charging/discharging.^25,26^ Second, the Cr^3+^ ion had a special electronic configuration (t^3^_2g_e^0^_g_).^48,49^ The introduction of Cr^3+^ into LDNs could promote electron capture and charge transfer.^48–50^ However, the Cr^3+^ ion itself did not take part in the faradaic redox reaction. When more Cr^3+^ ions were dropped into the LDNs, the capacitance performance decreased instead. Thus, a suitable Ni^2+^/Cr^3+^ ratio (2 : 1) was essential for achieving high capacitance.

### Electrochemical characterization of the asymmetric supercapacitor

**Fig. 7:**
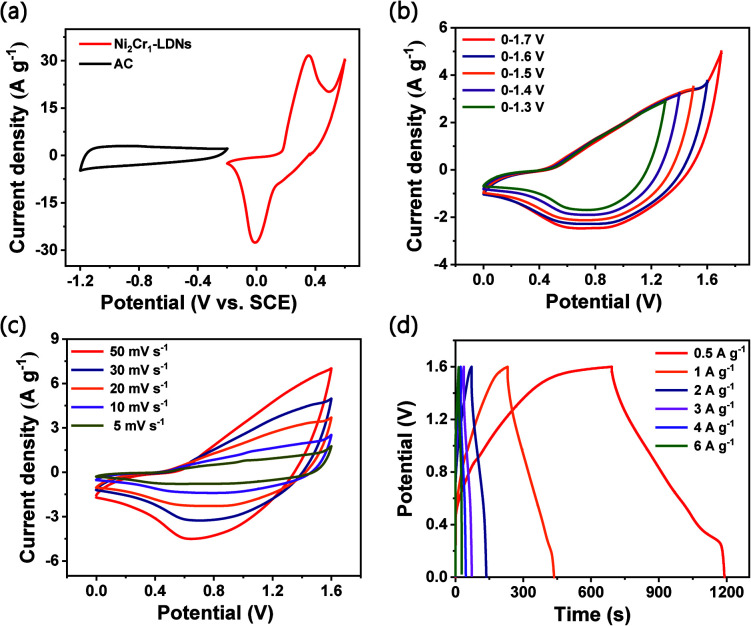
(a) CV curves of Ni_2_Cr_1_-LDNs and AC at a scan rate of 10 mV s^−1^, (b) CV curves of the ASC performed in different potential windows at 20 mV s^−1^, (c) CV curves at different scan rates from 5 to 50 mV s^−1^, and (d) GCD curves at different current densities.

An asymmetric supercapacitor (ASC) device was assembled to demonstrate the practical applications of the Ni_2_Cr_1_-LDN electrodes. The device wass denoted as the Ni_2_Cr_1_-LDN//AC ASC. The related electrochemical characteristics of the AC electrode in a 6 M KOH electrolyte are shown in Fig. S9.†Fig. 7a exhibits the CV curves of the Ni_2_Cr_1_-LDN electrode and AC electrode at a scan rate of 10 mV s^−1^. Fig. 7b shows the CV curves of Ni_2_Cr_1_-LDN//AC ASC in different voltage windows. Polarization occurred when the operating potential was higher than 1.6 V. Therefore, 0–1.6 V was selected as the suitable operating potential window of the Ni_2_Cr_1_-LDN//AC ASC. Fig. 7c exhibits the CV curves of the Ni_2_Cr_1_-LDN//AC ASC at scan rates from 5 to 50 mV s^−1^. They presented strong redox peaks, indicating the pseudocapacitive behaviour of the ASC. Fig. 7d shows the GCD curves of the Ni_2_Cr_1_-LDN//AC ASC at various current densities with a voltage range from 0 to 1.6 V, which also revealed the pseudocapacitive behaviour. The specific capacitance of the Ni_2_Cr_1_-LDN//AC ASC reached 155 F g^−1^ at 0.5 A g^−1^, and 49.5 F g^−1^ was retained at 6 A g^−1^ with a capacitance retention of 31.9%.

**Fig. 8:**
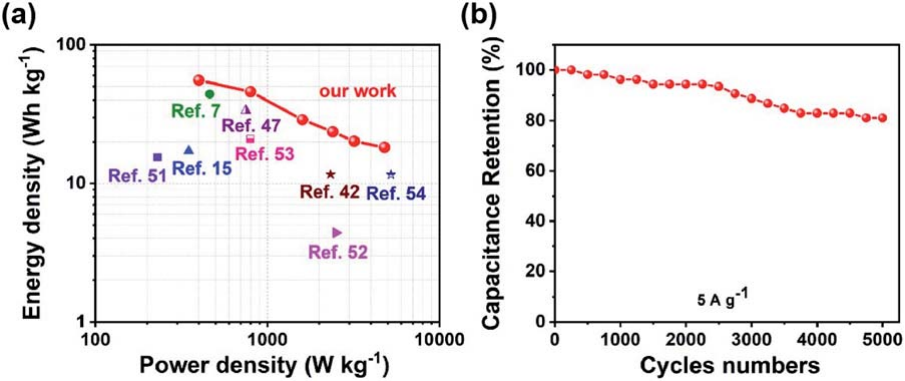
(a) Ragone plot related to the energy and power density of the ASC, (b) cycling performance of the ASC.

Fig. 8a shows the Ragone plots of the Ni_2_Cr_1_-LDN//AC ASC. The Ni_2_Cr_1_-LDN//AC ASC presented a maximum energy density of 55.33 W h kg^−1^ at 400 W kg^−1^. Besides, the energy density of the Ni_2_Cr_1_-LDN//AC ASC was maintained at 18.27 W h Kg^−1^, even at a power density of 4800 W kg^−1^. These values were comparable with or superior to those of most reported ASCs, such as NiFe LDHs/rGO/NF//MC (17.71 W h kg^−1^ at 348.49 W kg^−1^),^15^ NiAl-LDHs/rGO//AC (15.4 W h kg^−1^ at 230 W kg^−1^),^51^ CC@NiCo_2_Al-LDHs//CC@ZPC (44 W h kg^−1^ at 462 W kg^−1^),^7^ CoMn-LDHs//AC (4.4 W h kg^−1^ at 2500 W kg^−1^),^52^ NiCo-LDHs/graphene/nickel foam//AC (33.75 W h kg^−1^ at 750 W kg^−1^),^47^ NiMn-LDHs/PC//AC (11.65 W h kg^−1^ at 2330.16 W kg^−1^),^42^ NiAl-LDHs//AC (21 W h kg^−1^ at 800 W kg^−1^),^53^ and NiCo_2_O_4_//AC (11.6 W h kg^−1^ at 5220 W kg^−1^),^54^ shown in Fig. 8a. Moreover, the Ni_2_Cr_1_-LDN//AC ASC exhibited good cycling stability (Fig. 8b), with 81.1% of the capacitance retained, even after 5000 cycles.

## Conclusions

We have successfully synthesized thin NiCr-LDN nanoflakes for a supercapacitor with various Ni^2+^/Cr^3+^ ratios in one-step, efficient synthesis without hydrothermal treatment or extra exfoliation using organic solvents. These as-prepared LDH nanoflakes, with a 4–5 nm thickness, displayed increased contact area and enhanced capacitance and facilitated the diffusion of ions. The optimal Ni_2_Cr_1_-LDN (4.7 nm thickness) nanoflakes displayed an excellent specific capacitance of 1525 F g^−1^ at a current density of 2 A g^−1^, and a low charge transfer resistance of 0.26 Ω. Besides, the assembled Ni_2_Cr_1_-LDN//AC ASC delivered an outstanding energy density of 55.22 W h kg^−1^ at a power density of 400 W kg^−1^ and retained 81.1% of the original specific capacitance, even after 5000 cycles. This work provides a facile approach to efficiently synthesize LDH nanoflakes for energy storage.

## Conflicts of interest

There are no conflicts to declare.

## Supplementary Material

NA-002-D0NA00178C-s001
