# Supplementary material for: One-step facile synthesis of nickel–chromium layered double hydroxide nanoflakes for high-performance supercapacitors
Source: Nanoscale Adv. 2020 Apr 24;2(5):2099–105. doi: 10.1039/d0na00178c (PMC9419058; doi:10.1039/d0na00178c)
Supplement: NA-002-D0NA00178C-s001 [file NA-002-D0NA00178C-s001.pdf]

Electronic Supplementary Material (ESI) for Nanoscale Advances.
This journal is © The Royal Society of Chemistry 2020

Supporting Information

One-step facile synthesis of nickel-chromium layered double hydroxides nanoflakes for high-performance supercapacitors

Zuo Chen,‡[a] Hao Deng,‡[a] Man Zhang,[a] Zhiyu Yang,[a] Di Hu,[a] Yuchen Wang[a] and Kai Yan*[a, b]

[a] School of Environmental Science and Engineering, Sun Yat-sen University, 135 Xingang Xi Road, Guangzhou 510275, P. R. China

[b] Guangdong Provincial Key Laboratory of Environmental Pollution Control and Remediation Technology, Guangzhou 510275, P. R. China

‡These Authors contribute equally to this work.

* Corresponding author E-mail: yank9@mail.sysu.edu.cn

Cleaning of Ni foam

The nickel foam should be cleaned before being loaded with the active materials. The nickel foams were ultrasonic treated for 1 h in 1 M HCl, then washed with ethanol (10 mL) and deionized water (10 mL) for three times, respectively. Finally, the Ni foam was dried at 70 °C for 12 h in vacuum.

Table S1. Textural properties of Ni_1Cr_1-LDNs, Ni_2Cr_1-LDNs and Ni_3Cr_1-LDNs.

Simples	S_{BET} (m^2 g^{-1})	V_{total} (cm^3 g^{-1})	Size adsor (Å)
Ni_1Cr_1-LDNs	62.541	0.065	18.053
Ni_2Cr_1-LDNs	73.416	0.073	28.061
Ni_3Cr_1-LDNs	63.286	0.068	21.067

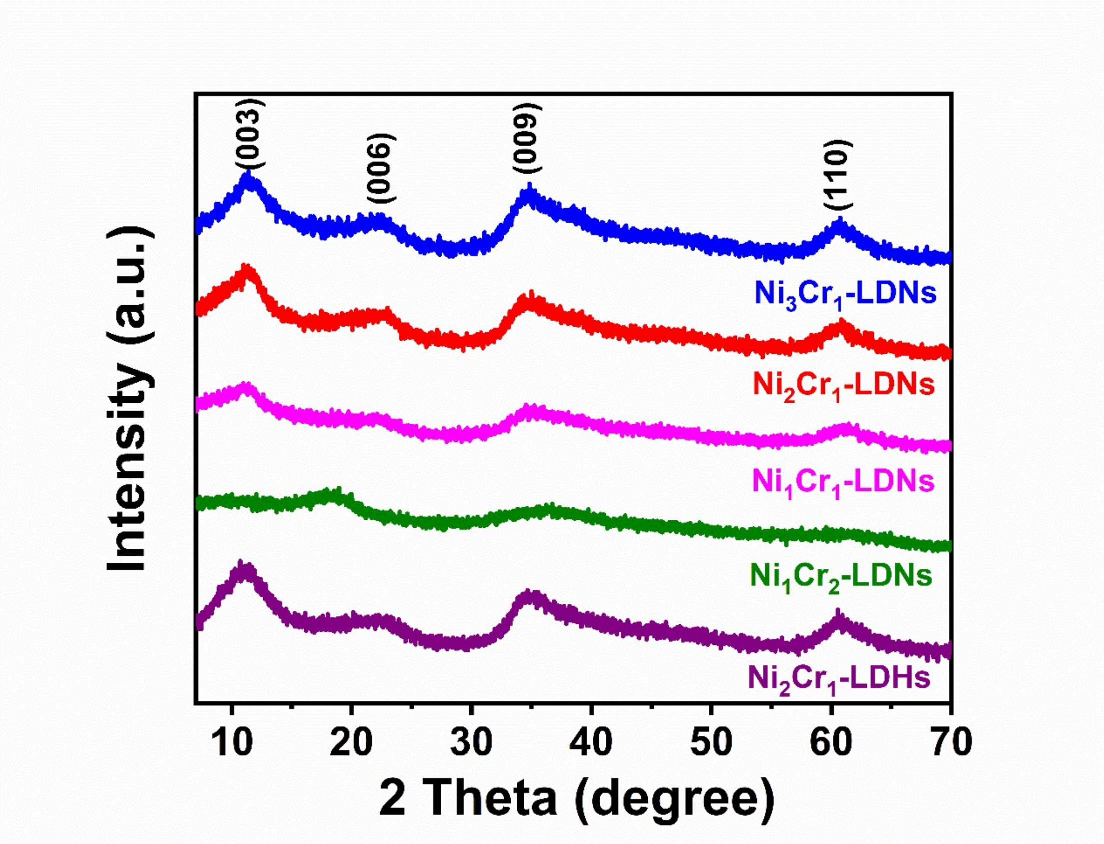

Fig. S1 XRD patterns of Ni_3Cr_1-LDNs, Ni_2Cr_1-LDNs, Ni_1Cr_1-LDNs, Ni_1Cr_2-LDNs and Ni_2Cr_1-LDHs.

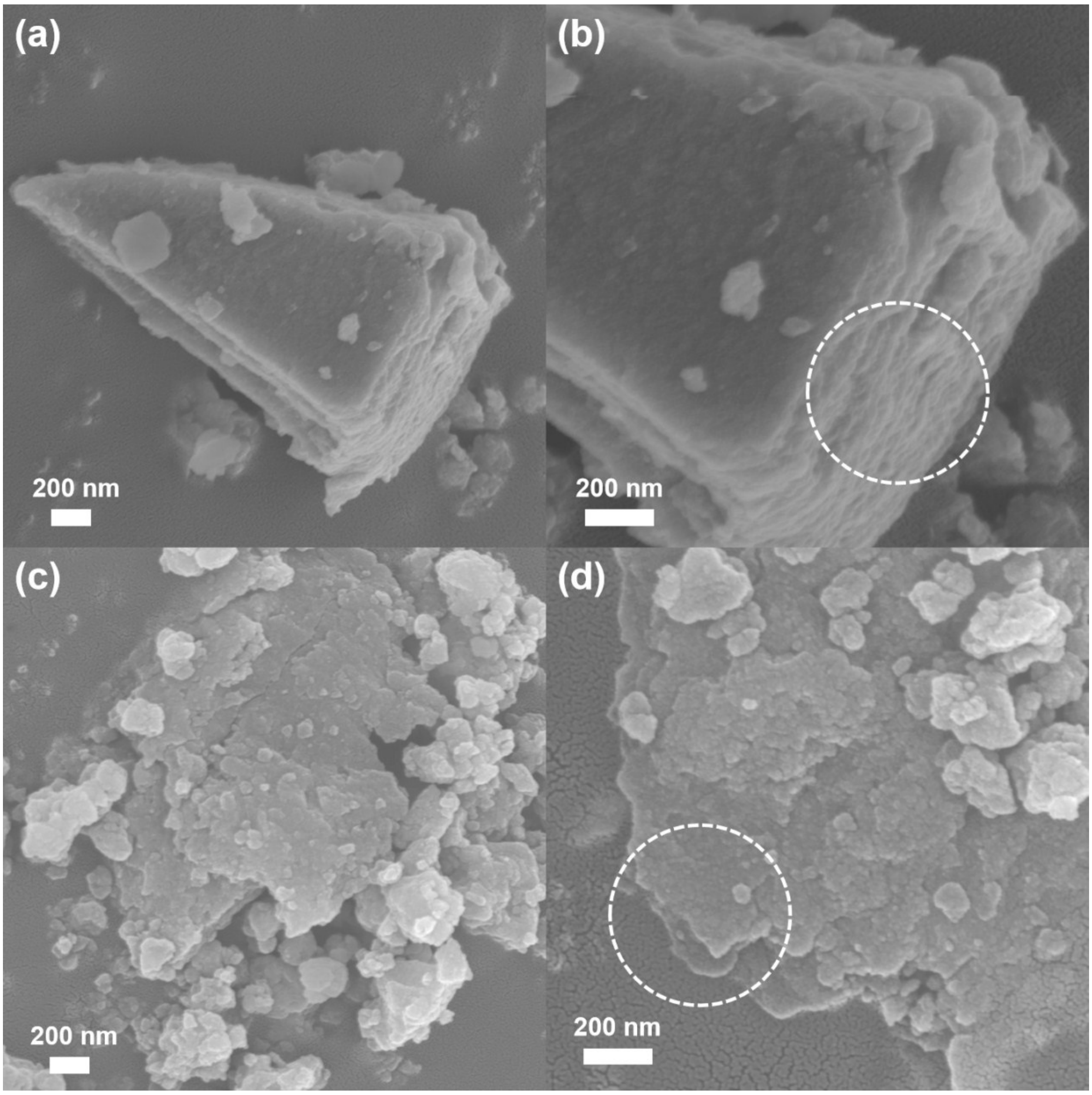

Fig. S2 SEM images of the synthesized Ni_2Cr_1-LDHs (a) and (b); Ni_2Cr_1-LDNs (c) and (d).

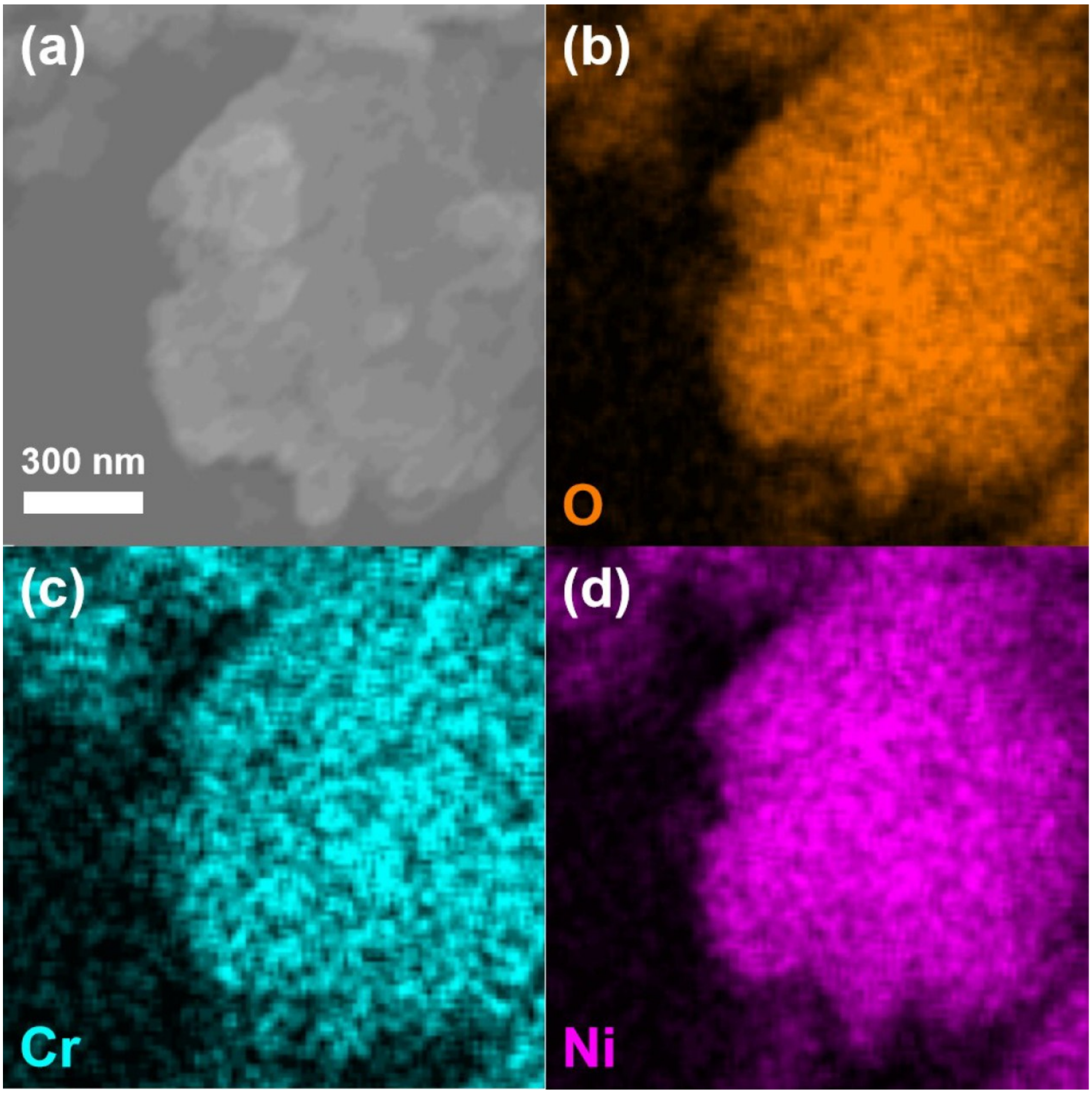

Fig. S3 SEM image of the Ni_2Cr_1-LDNs (a) and the corresponding EDS mapping of O (b), Cr (c) and Ni (d) elements.

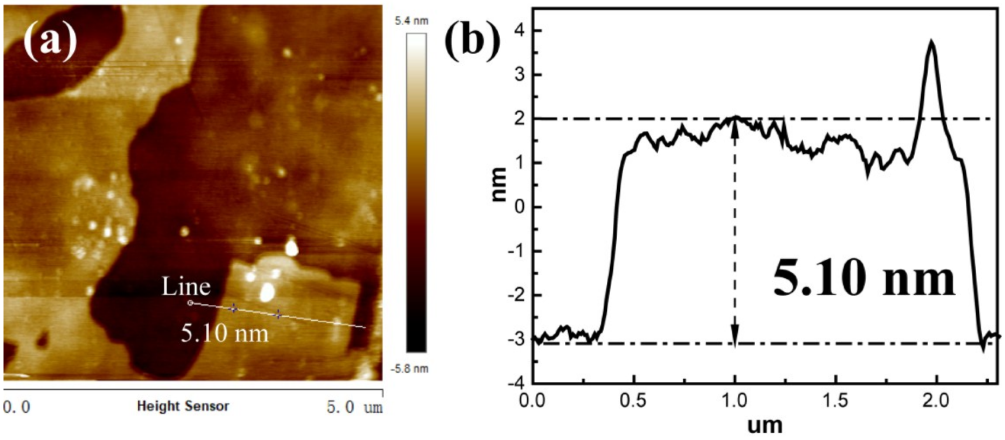

Fig. S4 (a) AFM image and (b) the corresponding height profile of Ni_2Cr_1-LDNs.

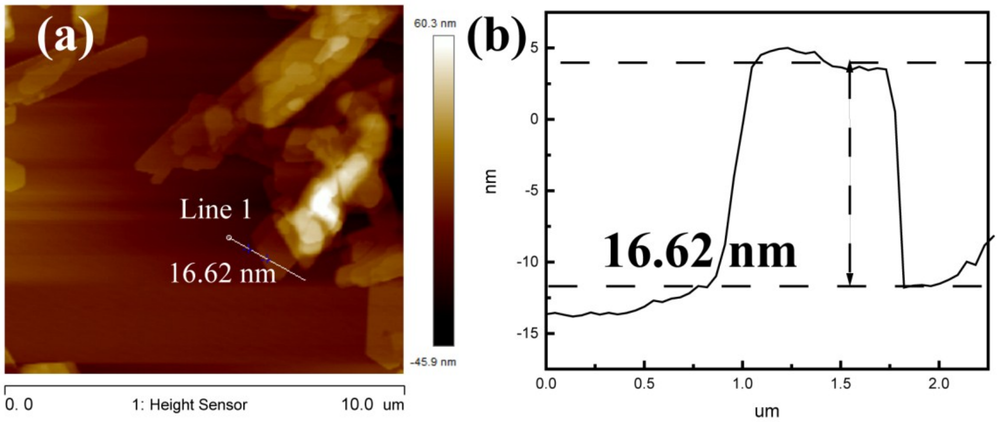

Fig. S5 (a) AFM image and (b) the corresponding height profile of Ni_1Cr_1-LDNs.

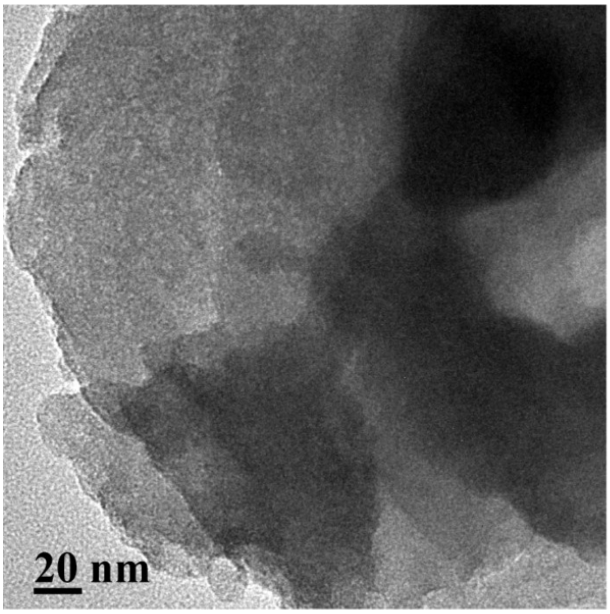

Fig. S6 Transmission Electron Microscopy image of Ni_1Cr_1-LDNs.

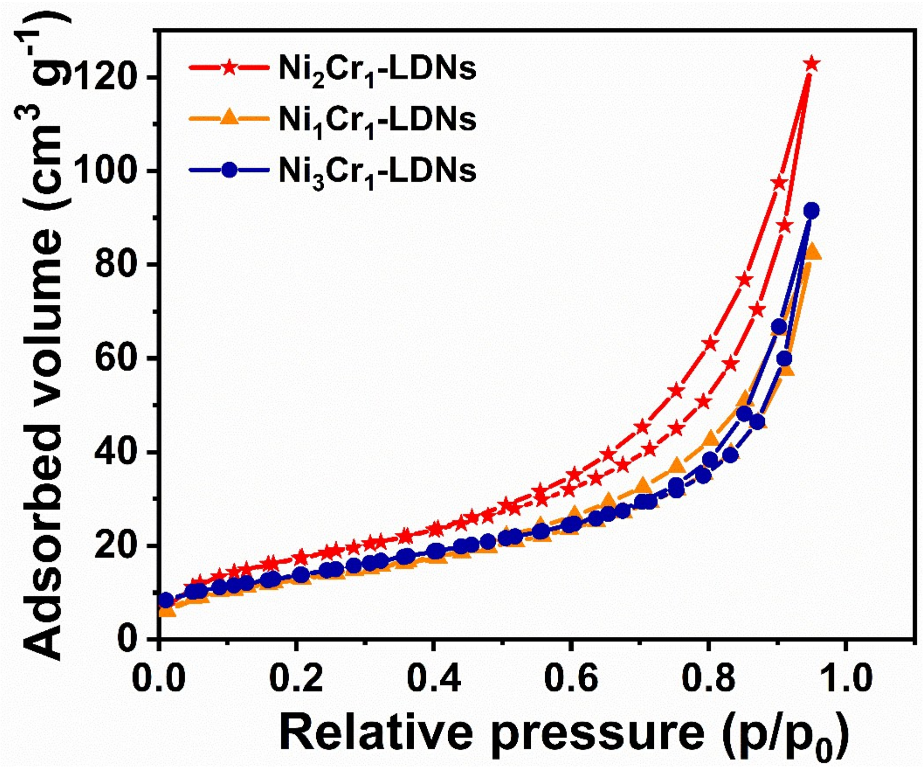

Fig. S7 N_2 adsorption/desorption isotherms of Ni_1Cr_1-LDNs, Ni_2Cr_1-LDNs and Ni_3Cr_1-LDNs.

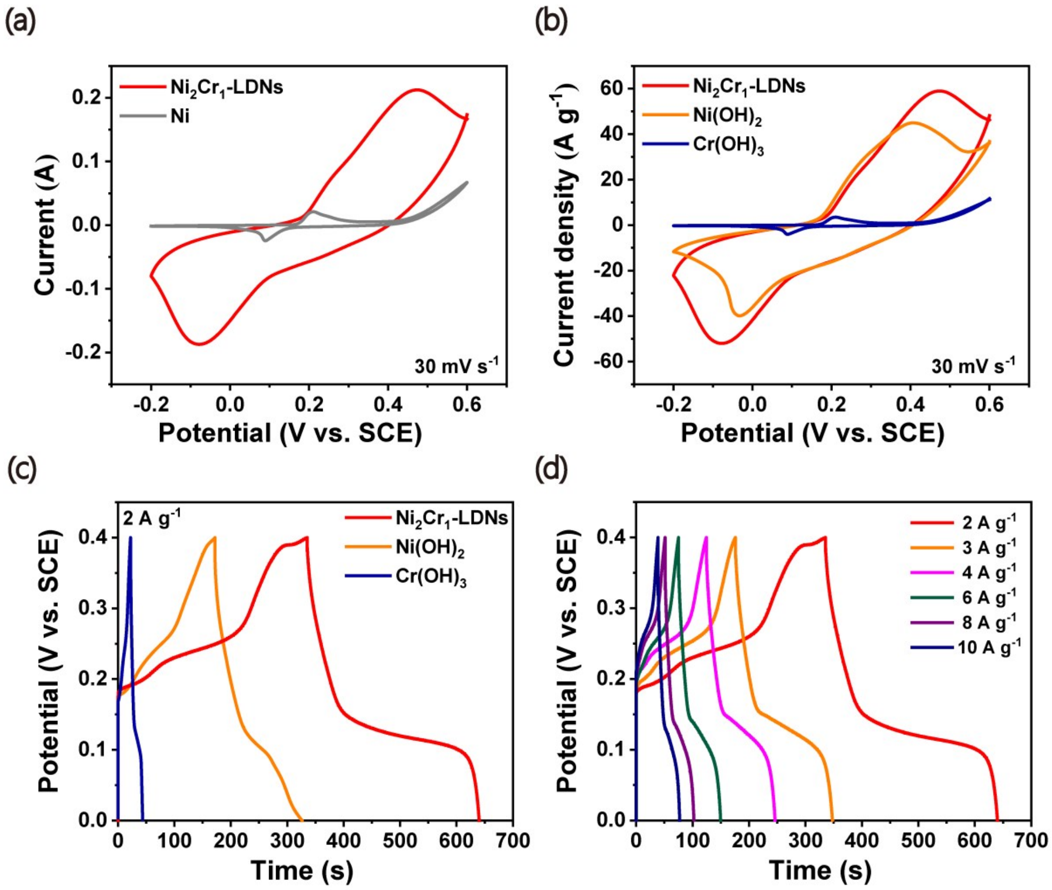

Fig. S8 (a) CV curves of Ni_2Cr_1-LDNs and pure nickel foam at the scan rate of 30 mV s^{-1} in 6 M KOH, (b) CV curves of Ni_2Cr_1-LDNs, $Ni(OH)_2$ and $Cr(OH)_3$ at the scan rate of 30 mV s^{-1} in 6 M KOH, (c) GCD curves of Ni_2Cr_1-LDNs, $Ni(OH)_2$ and $Cr(OH)_3$ at current densities of 2 A g^{-1}, (d) GCD curves of Ni_2Cr_1-LDNs at various current densities from 2 to 10 A g^{-1}.

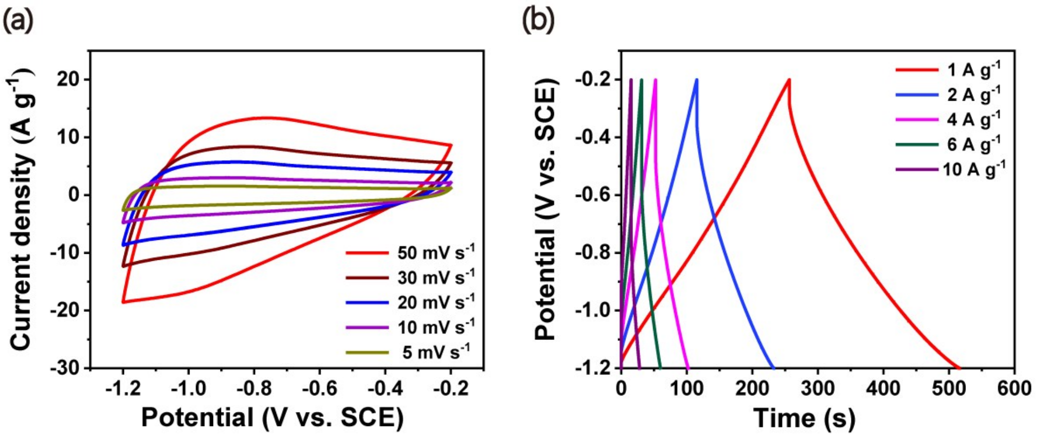

Fig. S9 (a) CV curves of AC at different scan rates from 5 to 50 mV s^{-1}, (b) GCD curves of AC at various current densities from 1 to 10 A g^{-1}.

For

$$\log k_s = a\log(1-a) + (1-a)\log a - \log\frac{RT}{nFv} - \frac{a(1-a)nF\Delta Ep}{2.303RT} \quad (5)$$

where the values of T, n, R, F and a is 298, 1, 8.314, 96500 and 0.42, respectively.
